# Annotations

**Published:** 1894-12-08

**Authors:** 


### ANNOTATIONS.

### How Sanitation is Done in Ireland.

"THEY do these things worse in Ireland." This was the burden of Dr. A. Roche's vaticination as president of the inaugural meeting of the Medical Society of the Catholic University Medical School in Dublin the other evening. Undoubtedly they do these sanitary things worse in Ireland; and what is the result? A high death-rate in a healthy country. With a country surrounded by the sea, almost without manufactures, with but few town populations, and with most of the people devoted to outdoor employments, Ireland has a death-rate not only higher than that of England or Scotland, but higher than those of continental countries whose populations are crowded thick together, and whose inhabitants work in close factories, on low wages, and seldom breathe the air of the open country or the sea. Why is this? "Want of education on the part of medical officers of health, and of sanitary inspectors; but still more want of interest on the part of local and Imperial authorities," says Dr. Roche. In Ireland the average medical officer of health is the dispensary doctor. The ordinary sanitary inspector is, Dr. Roche tells us, very commonly the man who has failed at everything else, and who is as fit for the teaching of Chinese as he is for sanitary inspection. But inasmuch as the medical officer of health and the sanitary inspector ordinarily constitute the whole of the administrative sanitary authority, it is obvious that, if both these officers are without adequate knowledge of their work, the work itself must of necessity fail to be done. It is not done; and Ireland is, by consequence, one of the most insanitary, if not quite the most insanitary, of all European countries. Dr. Roche, as is usual in Ireland, makes his appeal to the Imperial authorities. Ireland must, however, work out her own salvation in sanitary science if Imperial help is to be of any real and permanent service.

### Rolling of the Head.

INDIVIDUAL symptoms taken by themselves are proverbially fallacious. Nevertheless, it is often the case that the occurrence of some one symptom, even although not pathognomonic of a special morbid condition, may by its frequent association with it suggest its presence in cases where otherwise it might easily have been overlooked. This is markedly the case in infants who cannot even state the locality of their pain, and whose diseases have, therefore, to be diagnosed entirely by their objective symptoms. It is important, then, to recognise the meaning of that symptom which occurs so frequently in the course of so many of the febrile diseases of infancy, viz., rolling of the head. Dr. Woakes says this undoubtedly points to labyrinthine mischief. Here, then, we have a symptom generally associated in a vague sort of way with cerebral irritation, but which, to those who know, points to implication of the deeper structures of the ear, being, as Dr. Woakes points out, the infantile equivalent of vertigo in the adult. Without entering into anatomical reasons the "tip" is to examine and treat the ear whenever this symptom is present. We must recognise how frequently it happens that people die of what may be termed complications rather than of the disease they are thought to be suffering from. Children said to die of measles are really carried off by broncho-pneumonia; small-pox often kills by secondary sapræmia; and so when children suffering from teething, or the various exanthemata, especially from measles and scarlatina, sink into coma and convulsions, they die in reality, not from these ailments, but from cerebral inflammation. This process, however, reaches the brain through the ear, and rolling of the head means that it has already gone a long way on its course. Hence its importance. Among the other symptoms of a sharp febrile illness those of earache, the early signs of implication of the ear, are apt to be hidden and unnoticed, but with rolling of the head an immediate examination of the ears is necessary, for this is not an early sign, but a symptom of deep disease. In infants the meatus is very short, and the tympanum is accessible enough on the one condition that an anæsthetic is given. Thus it is as essential in regard to treatment as in regard to diagnosis to remember that rolling of the head means labyrinthine pressure.

### Compulsory Removal to Hospitals.

A CASE has been tried in the Scotch Courts which is of much interest to those who administer and to those who are under the jurisdiction of the Contagious Diseases Notification Act. The daughter, aged five and a half years, of an Aberdeen blacksmith, was the subject of an infectious disease, and was ordered to be removed to the Fever Hospital by the Lord Provost, Magistrates, and Town Council of the City of Aberdeen on their own authority, and without the warrant of a medical certificate from the proper medical official. Accordingly she was removed, in spite of a protest from the father, who had been advised by his own medical attendant that removal would be dangerous to life. The event justified the protest, for the little girl died at the hospital. The father thereupon commenced an action against the authorities, claiming £500 damages. His contention was that the removal to the hospital, together with the neglect of sufficient precautions in removal and inadequate treatment at the hospital had caused or accelerated the fatal issue. He further contended that the town authorities had no such powers as they claimed, they not having been armed by public medical warrant. One of the lower courts decided in favour of the father's plea, and a second in favour of the defenders. But the first Division of the Court of Session upheld the original decision, and sent the case for trial by a common jury. It is the ground of their lordships' decision which is of so much importance to all who are interested in the Contagious Diseases Notification Act; that is, to all classes of the public. The Lord Provost and Town Council had caused the removal of the child without a certificate from the Medical Officer of Health. Therein the High Court decided that they had acted *ultra vires*. Moreover, in so conducting the removal as to expose the child to increased risk, and in their neglect to ensure her against inadequate treatment at the hospital, they had failed in those common and obvious duties of humanity which even town councils and magistrates must be compelled to respect. The decision of the High Court is very important. It establishes the precedent that fever patients must not be removed to State hospitals on lay, but only on recognised medical authority; and also that, when they are removed, the removing public body must ensure first that no added risks shall accompany the removal, and, secondly, that adequate medical provision shall be available at the isolation hospital. All this is exactly as it should be. The Contagious Diseases Notification Act is designed for the protection of the public which is still healthy. But in protecting the public, it is obvious that its administration must not be such as to increase the risks of the sick. The State must protect both the well and the sick as far as it can, but especially the sick.

